# The PqsE-RhlR Interaction Regulates RhlR DNA Binding to Control Virulence Factor Production in *Pseudomonas aeruginosa*

**DOI:** 10.1128/spectrum.02108-21

**Published:** 2022-01-12

**Authors:** Kayla A. Simanek, Isabelle R. Taylor, Erica K. Richael, Erica Lasek-Nesselquist, Bonnie L. Bassler, Jon E. Paczkowski

**Affiliations:** a Department of Biomedical Sciences, School of Public Health, University at Albany, Albany, New York, USA; b Department of Molecular Biology, Princeton University, Princeton, New Jersey, USA; c Division of Genetics, Wadsworth Center, New York State Department of Health, Albany, New York, USA; d Howard Hughes Medical Institute, Chevy Chase, Maryland, USA; Emory University School of Medicine

**Keywords:** protein-protein interactions, quorum sensing, transcriptional regulation, virulence factors

## Abstract

Pseudomonas aeruginosa is an opportunistic pathogen that causes disease in immunocompromised individuals and individuals with underlying pulmonary disorders. P. aeruginosa virulence is controlled by quorum sensing (QS), a bacterial cell-cell communication mechanism that underpins transitions between individual and group behaviors. In P. aeruginosa, the PqsE enzyme and the QS receptor RhlR directly interact to control the expression of genes involved in virulence. Here, we show that three surface-exposed arginine residues on PqsE comprise the site required for interaction with RhlR. We show that a noninteracting PqsE variant [PqsE(NI)] possesses catalytic activity, but is incapable of promoting virulence phenotypes, indicating that interaction with RhlR, and not catalysis, drives these PqsE-dependent behaviors. Biochemical characterization of the PqsE-RhlR interaction coupled with RNA-seq analyses demonstrates that the PqsE-RhlR complex increases the affinity of RhlR for DNA, enabling enhanced expression of genes encoding key virulence factors. These findings provide the mechanism for PqsE-dependent regulation of RhlR and identify a unique regulatory feature of P. aeruginosa QS and its connection to virulence.

**IMPORTANCE** Bacteria use a cell-cell communication process called quorum sensing (QS) to orchestrate collective behaviors. QS relies on the group-wide detection of molecules called autoinducers (AI). QS is required for virulence in the human pathogen Pseudomonas aeruginosa, which can cause fatal infections in patients with underlying pulmonary disorders. In this study, we determine the molecular basis for the physical interaction between two virulence-driving QS components, PqsE and RhlR. We find that the ability of PqsE to bind RhlR correlates with virulence factor production. Since current antimicrobial therapies exacerbate the growing antibiotic resistance problem because they target bacterial growth, we suggest that the PqsE-RhlR interface discovered here represents a new candidate for targeting with small molecule inhibition. Therapeutics that disrupt the PqsE-RhlR interaction should suppress virulence. Targeting bacterial behaviors such as QS, rather than bacterial growth, represents an attractive alternative for exploration because such therapies could potentially minimize the development of resistance.

## INTRODUCTION

The opportunistic human pathogen Pseudomonas aeruginosa infects immunocompromised individuals and those with underlying pulmonary disorders. According to the Centers for Disease Control and Prevention, Enterococcus faecium, Staphylococcus aureus, Klebsiella pneumoniae, Acinetobacter baumannii, P. aeruginosa, and Enterobacter spp., collectively known as ESKAPE pathogens, represent a significant threat to human health because they are pathogenic and commonly multidrug resistant (1). Therefore, new effective treatments are urgently needed. In the case of P. aeruginosa, virulence is driven by quorum sensing (QS), a cell-to-cell communication process that relies on the production, release, accumulation, and detection of extracellular signal molecules called autoinducers (AI) (2–7). QS facilitates synchronous, population-wide alterations in the expression of genes that underpin collective behaviors, such as biofilm formation and virulence factor production (8, 9).

Two LuxR/LuxI-type receptor/synthase pairs, LasR/LasI and RhlR/RhlI, are central to P. aeruginosa QS. LasR/LasI resides at the top of the hierarchy (10–13). LasI synthesizes the AI *N*-3-oxo-dodecanoyl-l-homoserine lactone (3OC_12_HSL), which binds to LasR (7, 14). Binding of 3OC_12_HSL stabilizes and activates LasR, which is a transcription factor (15, 16). Thus, ligand binding promotes LasR DNA binding and the activation of transcription of the genes in its regulon, among which are *rhlI* and *rhlR* (12, 17). RhlI synthesizes the AI *N*-butyryl-homoserine lactone (C_4_HSL), which binds to its partner receptor RhlR (18, 19). RhlR, like LasR, is a transcription factor, and the RhlR:C_4_HSL complex launches a second wave of QS target gene expression (20). Each ligand-bound receptor activates the expression of the gene encoding its respective synthase. These so-called autoinduction feedback loops ramp up AI production and, since newly made AI further activates the partner receptor, these loops increase target gene expression (14, 21, 22).

The third P. aeruginosa QS circuit, and a focus of the present work, is called the Pseudomonas quinolone signaling (PQS) system and is comprised of the *pqsABCDE* operon, *pqsH*, and *pqsR* (23–26). PqsABCD are responsible for biosynthesis of a molecule called HHQ (4-hydroxy-2-heptylquinolone) and PqsH is required to convert HHQ into the AI called PQS (2-heptyl-3-hydroxy-4-quinolone) (27). PqsR, the PQS receptor, is activated upon binding either HHQ or PQS, with PQS understood to be the primary ligand (23, 28). The PqsR:PQS complex controls transcription of genes involved in virulence factor production and biofilm formation (29). Analogous to the above, there is an autoinduction feedback loop: the PqsR:PQS complex activates transcription of *pqsABCDE*, promoting increased PQS synthesis, increased PQS-mediated activation of PqsR, and increased transcription of target genes. Additionally, expression of *pqsABCDE*, *pqsH*, and *pqsR* is regulated by both the Las and Rhl QS systems (30).

The role PqsE plays in PQS QS is mysterious (24, 28, 31–35). *pqsE* is the final gene in an operon with genes that are required for PQS biosynthesis. Curiously, however, a Δ*pqsE* P. aeruginosa mutant produces wild-type (WT) levels of PQS (33, 36). *In vitro*, PqsE converts 2-aminobenzoylacetyl-CoA (2-ABA-CoA) to 2-aminobenzoyl acetate (2-ABA) (31). If, in P. aeruginosa, this reaction is on the pathway to PQS production, some other thioesterase(s) must perform this catalytic step in the Δ*pqsE* mutant. Also puzzling is that a Δ*pqsE* mutant does not produce the QS-controlled virulence factor called pyocyanin (28). However, supplementation of this mutant with PQS precursors or the PQS AI does not complement the defect. Finally, *pqsE* is essential for P. aeruginosa virulence in animal models, demonstrating that PqsE performs a required pathogenicity function (32). Together, the above findings suggest that the role PqsE plays in P. aeruginosa virulence is distinct from its function as an enzyme.

We recently demonstrated that pyocyanin production is controlled through a physical interaction between RhlR and PqsE (33, 36–38). Specifically, we showed that PqsE variants which mimic the inhibitor-bound state of PqsE disrupt the interaction with RhlR and attenuate pyocyanin production. In the earlier work, we hypothesized that PqsE interaction with RhlR enhances RhlR affinity for DNA. Here, we determined the surface residues on PqsE responsible for interaction with RhlR and characterized the role of the PqsE-RhlR complex in P. aeruginosa QS. Using structure-guided mutagenesis, we generated a triple-variant PqsE protein (R243A/R246A/R247A) which abolished the PqsE-RhlR interaction. We showed that introduction of this variant into P. aeruginosa eliminates pyocyanin production while the purified PqsE variant possesses catalytic activity *in vitro*. Thus, the mutations establish a putative binding site for RhlR that is distinct from the catalytic site, separating the two apparent PqsE functions. We used DNA gel shift analyses to demonstrate that PqsE binding to RhlR increases RhlR affinity for promoter DNA. RNA-seq analyses of strains harboring PqsE variants with differing abilities to interact with RhlR showed that levels of PqsE-RhlR complex formation *in vitro* correlate with the ability of P. aeruginosa to properly regulate RhlR-dependent genes *in vivo*. We conclude that binding of PqsE to RhlR is primarily through an α-helix containing R243/R246/R247. It is the PqsE-RhlR interaction, and not PqsE-driven catalysis, that underpins the regulation of RhlR by PqsE and, in turn, controls the production of pyocyanin and other important P. aeruginosa virulence factors.

## RESULTS

### An arginine-rich surface-exposed α-helix is required for PqsE interaction with RhlR.

**FIG 1:**
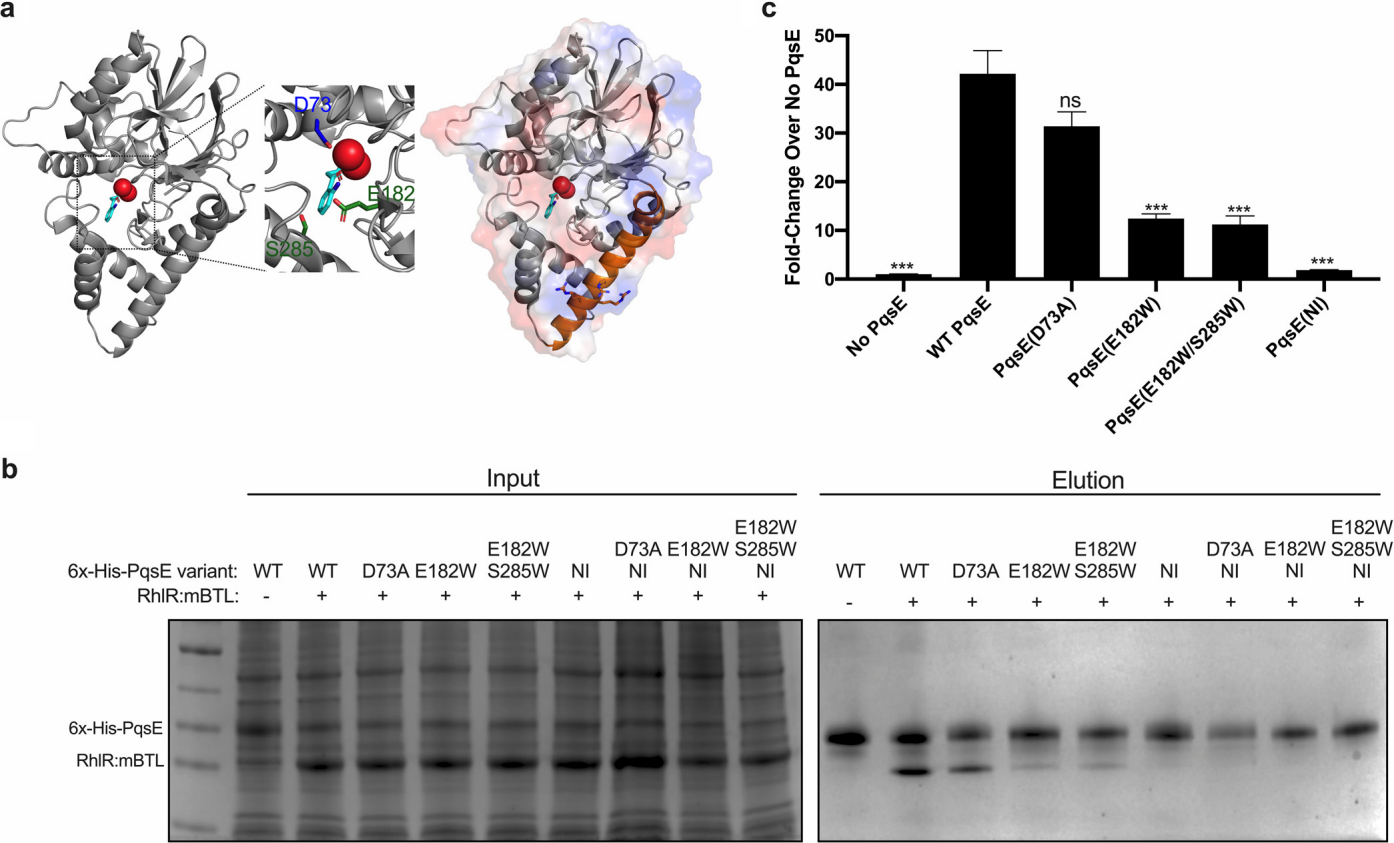
Mutational analysis of PqsE reveals the PqsE-RhlR interface. (a) Left: structure of PqsE (gray) bound to 2-ABA (cyan) coordinated by two Fe^2+^ ions (red) (PDB: 5HIO). Center inset*:* close-up view of the catalytic pocket highlighting residues identified as being important for catalysis (D73; blue), and to mimic inhibitor binding (S285 and E182; green). Right: surface representation (red = negative charge, blue = positive charge) overlay on the structure of PqsE (gray), highlighting α-helix 5 containing residues R243, R246, and R247 (orange). (b) SDS-PAGE of cell lysates before (Input) and after (Elution) affinity purification on Ni-NTA resin. Shown are WT and variant 6x-His-PqsE-containing lysates which had been combined with lysate containing (+) or lacking (-) RhlR:mBTL. In all affinity purification experiments, RhlR does not carry any tag. (c) Bioluminescence output from E. coli carrying *rhlR*, p*rhlA-luxCDABE*, and the designated *pqsE* alleles on pACYC184 in the presence of 500 nM C_4_HSL, with bioluminescence normalized to the OD_600_ of the cultures. Bars represent 2 biological replicates performed in technical triplicate. Error bars represent standard deviations of the means of biological replicates. Unpaired *t* tests compared the light produced by each strain to that produced by the strain with WT PqsE. *P* values: ns, ≥ 0.05; ***, <0.001.

We showed previously that when PqsE residue E182 or E182 together with S285 are substituted with tryptophan residues, the PqsE protein mimics the catalytically inhibited state and, moreover, the ability of PqsE to interact with RhlR is disrupted (37). E182 and S285 are in the PqsE catalytic pocket along with the D73 residue which is essential for catalysis (Fig. 1a; E182 and S285 are shown in green, D73 is shown in blue). The PqsE structure shows that while E182 and S285 are buried, they abut a surface exposed arginine-rich α-helix, (α-helix 5) which contains residues 227 to 255 (Fig. 1a; the surface exposed α-helix 5 harboring R243, R246, and R247 is shown in orange). We hypothesized that the arginine-rich α-helix could directly interact with RhlR and that perhaps this helix is perturbed in the PqsE(E182W) and PqsE(E182W/S285W) mutants. To test this possibility, we mutated R243, R246, and R247 to alanine residues and performed affinity purification analyses to assess interaction with RhlR. In this assay, PqsE is 6x-His-tagged and used as bait in the affinity purification, and RhlR is bound to the synthetic ligand meta-bromo-thiolactone (mBTL), which we have previously used to activate, solubilize, and purify RhlR (34). We call this complex RhlR:mBTL. As a control, we used PqsE(D73A), which, while lacking catalytic activity, interacts like WT PqsE with RhlR:mBTL (Fig. 1b). Throughout this work, all purified PqsE proteins contain N-terminal 6x-His tags. To simplify the nomenclature, we do not explicitly write this throughout the main text. Consistent with our recent findings, PqsE(E182W) and PqsE(E182W/S285W) both showed impaired binding to RhlR:mBTL (Fig. 1b). Strikingly, PqsE(R243A/R246A/R247A) showed a complete lack of interaction with RhlR:mBTL (Fig. 1b). In Fig. 1b and from here forward, we refer to PqsE(R243A/R246A/R247A) as PqsE(NI) for “PqsE Non-Interacting.” Our results with PqsE(NI) suggest that we have pinpointed the binding interface between PqsE and RhlR. Indeed, interaction of PqsE(D73A), PqsE(E182W), and PqsE(E182W/S285W) with RhlR:mBTL was abolished when the PqsE(NI) amino acid substitutions were introduced (Fig. 1b, PqsE D73A/NI, PqsE E182W/NI and PqsE E182W/S285W/NI). These findings are consistent with a model in which the PqsE catalytic pocket can allosterically influence the α-helix 5-mediated interface between RhlR and PqsE.

To probe the consequences of RhlR-PqsE complex formation on RhlR-dependent activation of gene expression, we used a recombinant Escherichia coli system in which *rhlR* expression is driven by the pBAD promoter and *pqsE* is constitutively expressed from the *lac* promoter (37, 39). The natural AI for RhlR, C_4_HSL is supplied exogenously to activate RhlR. We call this complex RhlR:C_4_HSL. Transcriptional output is assessed by the production of light from *luxCDABE* (luciferase) driven by the RhlR:C_4_HSL-controlled *rhlA* promoter. Inclusion of PqsE in this assay is known to enhance RhlR:C_4_HSL activation of *rhlA* expression (33, 37). Indeed, in the presence of 500 nM C_4_HSL, light production was 42-fold higher in the strain carrying RhlR:C_4_HSL and PqsE compared to the strain lacking PqsE (Fig. 1c). Light output correlated with the ability of the PqsE variants to interact with RhlR. Specifically, compared to the strain lacking PqsE, introduction of PqsE(D73A) increased light production to nearly the same level as when WT PqsE was present. This result is consistent with the ability of PqsE(D73A) to interact with RhlR similarly to WT PqsE (Fig. 1b). PqsE(E182W) and PqsE(E182W/S285W) each drove ∼11-fold higher light production than that from the no-PqsE control strain, again, consistent with the diminished ability of these variants to interact with RhlR compared to WT PqsE and PqsE(D73A), as shown in Fig. 1b. In contrast, the presence of the PqsE(NI) variant failed to increase light production above that of the control strain lacking PqsE (i.e., within 2-fold, Fig. 1c). All PqsE variants were produced to similar levels in the E. coli recombinant strain (Fig. S1 in the supplemental material). These results indicate that PqsE and RhlR:C_4_HSL interact to activate transcription and, given that these are the only P. aeruginosa components present in our E. coli system, suggest that PqsE likely enhances the affinity of RhlR:C_4_HSL for promoter DNA. We return to this point below.

### PqsE enzyme activity is not affected by interaction with RhlR.

**FIG 2:**
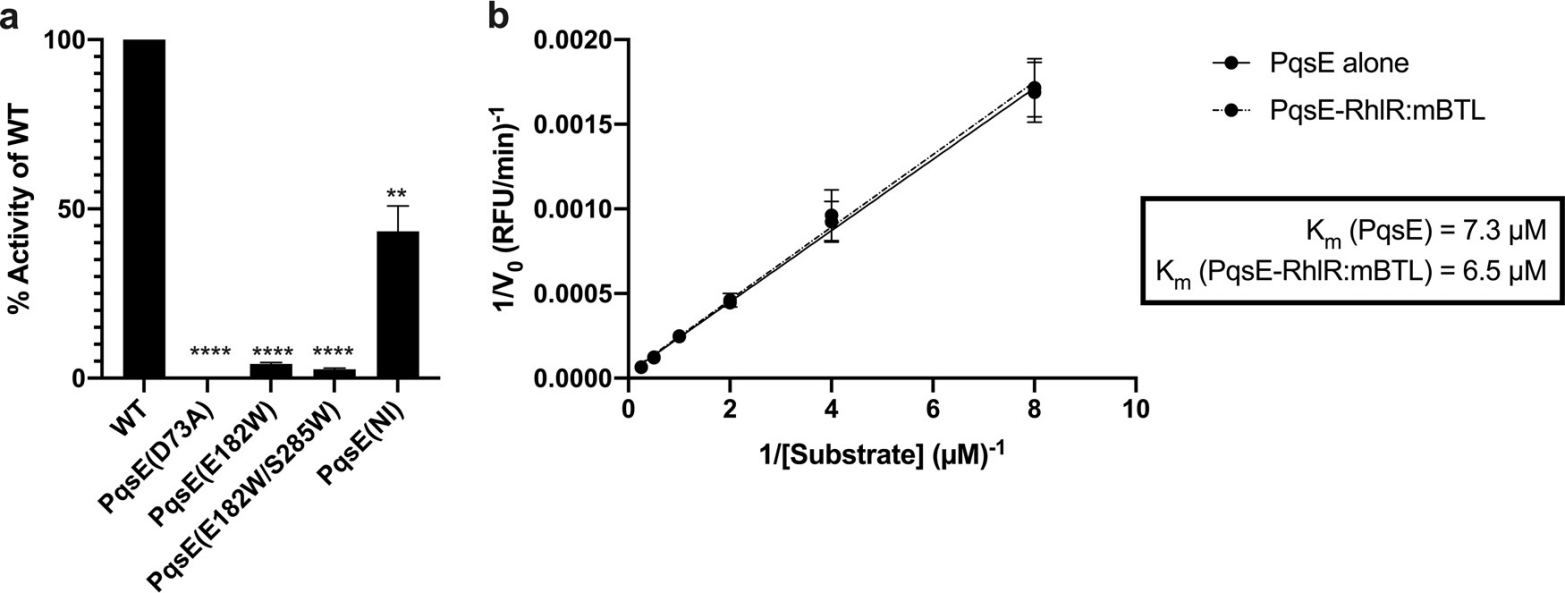
RhlR binding to PqsE does not affect PqsE catalytic function. (a) Catalytic activity of the designated purified PqsE proteins measured in terms of hydrolysis of MU-butyrate. Rate of hydrolysis by each protein is reported as percent activity compared to that of WT PqsE. Bars represent 3 independent experiments performed in technical triplicate. Error bars depict standard deviations of the means for each independent experiment. Unpaired *t* tests compared initial rates obtained for each PqsE variant to that for WT PqsE. *P* values: ns, ≥ 0.05; **, <0.01; ****, <0.0001. (b) Catalytic activity of PqsE and PqsE-RhlR:mBTL was measured for the designated concentrations of the MU-butyrate substrate, and Lineweaver-Burk plots were generated to determine kinetic parameters for PqsE alone (solid line) and for PqsE:RhlR:mBTL (dashed line).

The three arginine residues that are critical for PqsE to interact with RhlR reside on the surface of PqsE, a location distant from the buried active site. Therefore, these three residues are not predicted to play a direct role in PqsE catalytic function. To verify this notion, we used the synthetic substrate, 4-methylumbelliferyl butyrate (MU-butyrate), to quantify PqsE(NI) enzyme activity. As controls, we assayed WT PqsE and the catalytically inactive PqsE(D73A) variant. We compared these activities to the two inhibitor mimetic PqsE variants, PqsE(E182W) and PqsE(E182W/S285W). WT PqsE readily hydrolyzed MU-butyrate, PqsE(D73A) had no measurable enzyme activity, and the inhibitor mimetic variants were severely impaired, exhibiting less than 5% of WT activity (Fig. 2a). The PqsE(NI) protein, by contrast, displayed ∼40% of the activity of WT PqsE. In this case, somewhat reduced hydrolytic capacity is not entirely surprising given the lower stability of the PqsE(NI) protein [*T_m_* of PqsE(NI) = 62.3°C] compared to that of WT PqsE (*T_m_* of WT PqsE = 67.6°C; Fig. S2a).

Our next goal was to determine whether interaction with RhlR affects PqsE catalytic function. We purified WT PqsE and WT PqsE in complex with RhlR:mBTL. The concentrations of PqsE were normalized according to SDS-PAGE analysis (Figure S2b) and the MU-butyrate substrate was used to measure hydrolytic activity. PqsE in complex with RhlR:mBTL exhibited nearly identical enzyme kinetics as PqsE alone (Fig. 2b). Thus, binding to RhlR does not affect PqsE catalytic activity.

### The PqsE-RhlR interaction controls pyocyanin production.

**FIG 3:**
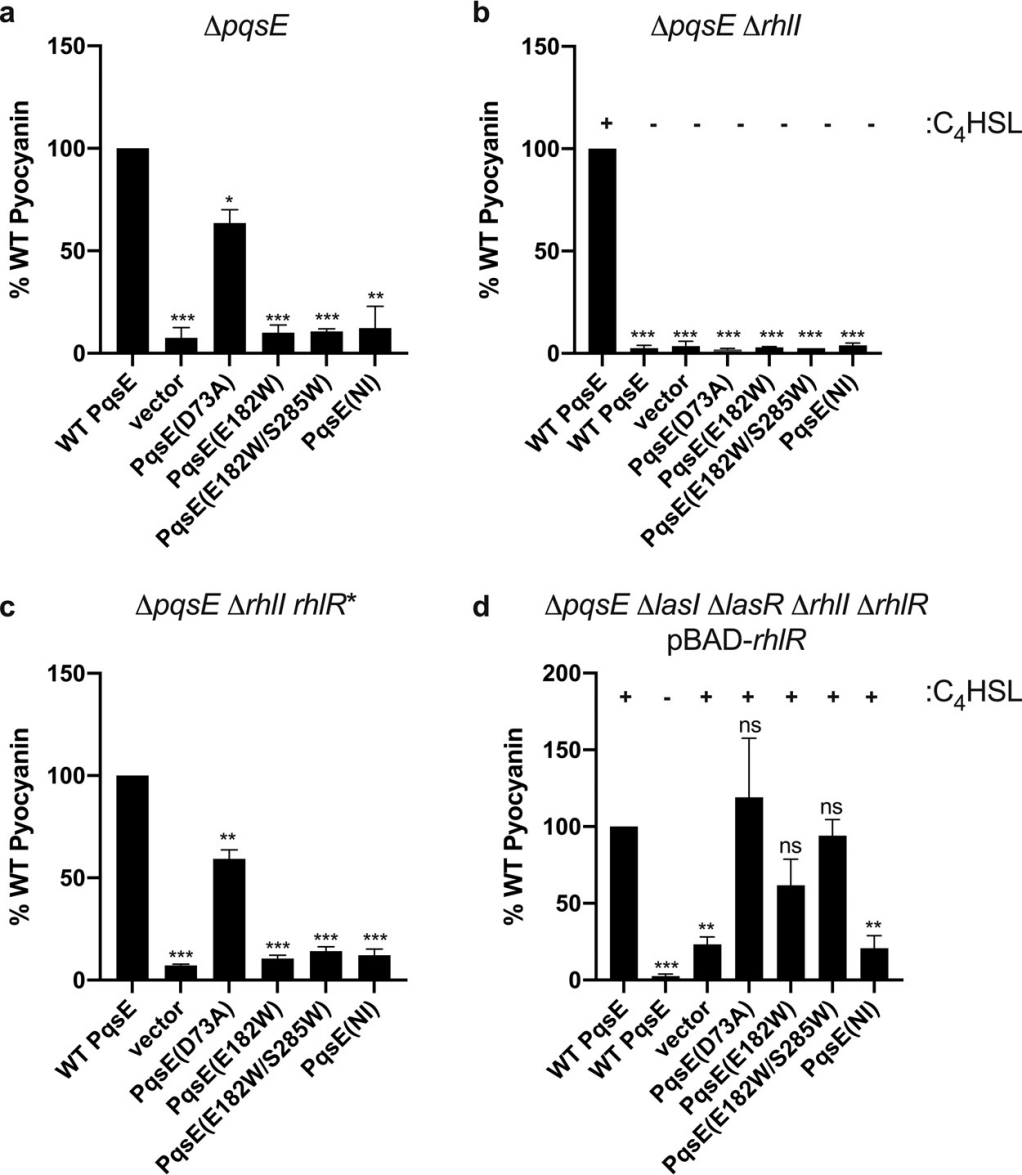
Strains containing PqsE variants that disrupt the PqsE-RhlR:C_4_HSL interface exhibit attenuated pyocyanin production. Pyocyanin production from P. aeruginosa strains carrying plasmid-produced PqsE variants, normalized to strains carrying plasmid-produced WT PqsE in (a) Δ*pqsE*, (b) Δ*pqsE* Δ*rhlI*, (c) Δ*pqsE* ΔrhlI rhlR* (rhlR* encodes a C_4_HSL-independent RhlR variant expressed from its native promoter), and (d) Δ*pqsE* Δ*lasI* Δ*lasR* Δ*rhlI* Δ*rhlR* with *rhlR* expressed from the pBAD promoter. C_4_HSL was supplied where indicated (+), and (-) designates that C_4_HSL was not added. Bars represent 3 biological replicates. Two technical replicates were performed and averaged for each biological replicate. Error bars represent standard deviations of the means of biological replicates. Unpaired *t* tests compared pyocyanin production from each strain to that produced by the strain with WT PqsE, as shown in each graph. *P* values: ns, ≥ 0.05; *, <0.05; **, <0.01; ***, <0.001; ****, <0.0001.

To understand what role the PqsE-RhlR interaction plays *in vivo* in P. aeruginosa, we assayed the ability of each of the PqsE variants to promote the production of the virulence factor pyocyanin. Both PqsE and RhlR are required for pyocyanin production (28, 32). WT *pqsE* and the *pqsE* mutants were cloned onto pUCP18 under the *lac* promoter. We introduced each construct into Δ*pqsE* P. aeruginosa. The strains carrying WT PqsE and PqsE(D73A) produced nearly the same amount of pyocyanin. In contrast, all of the strains harboring PqsE variants that exhibited impaired interaction with RhlR *in vitro* failed to produce pyocyanin *in vivo* (Fig. 3a). Thus, the interaction between PqsE and RhlR appears to be critical for pyocyanin production. To verify our strategy, we introduced each of the *pqsE* mutant genes onto the P. aeruginosa chromosome at the native site. All of the PqsE variant proteins were produced to similar levels as in WT PqsE (Fig. S3a) and their pyocyanin production profiles mirrored those in which the *pqsE* alleles were expressed from a plasmid (compare data in Fig. S3b to that in Fig. 3a). Thus, important for the work presented below, we can use plasmid-expressed *pqsE* to investigate the PqsE-RhlR complex in P. aeruginosa.

Distinguishing the individual functions of PqsE and the RhlI AI synthase in driving RhlR activity is complicated by the need for the RhlI-produced C_4_HSL molecule to stabilize and activate RhlR *in vivo*. RhlR* is a ligand-independent, constitutively active RhlR variant which contains three stabilizing hydrophobic amino acid substitutions in the ligand binding pocket (34). We hypothesized that we could exploit RhlR* to disentangle the role PqsE plays from that played by RhlI and C_4_HSL in RhlR activation of *in vivo* gene expression, again using pyocyanin production as our measure. Thus, we assessed pyocyanin production in Δ*pqsE* Δ*rhlI* and Δ*pqsE* ΔrhlI rhlR* strains (Fig. 3b and c). None of the PqsE variants, including WT PqsE, enabled pyocyanin production in the Δ*pqsE* Δ*rhlI* strain, presumably because *in vivo*, RhlR is inactive in the absence of the C_4_HSL ligand (Fig. 3b). In the context of the RhlR* allele, the strains carrying WT PqsE and PqsE(D73A) produced pyocyanin, while the strains harboring the three PqsE variants that were defective in interacting with RhlR did not (Fig. 3c). The ability of RhlR* to physically interact with WT PqsE and the different PqsE variants was indistinguishable from that of WT RhlR:mBTL (compare results in Fig. S4a to those in Fig. 1b). Together, these data show that *in vivo* pyocyanin production relies on the RhlR interaction with PqsE, and that the role of RhlI is to produce the C_4_HSL ligand required to activate the RhlR protein.

To determine if, in the absence of other key P. aeruginosa QS components, the PqsE-RhlR:C_4_HSL interaction is sufficient to promote pyocyanin production, we performed the pyocyanin assay in a strain in which we had deleted *lasR*, *lasI*, *rhlR*, *rhlI*, and *pqsE*. We reintroduced *rhlR* under the control of the pBAD promoter, supplied exogenous C_4_HSL to activate RhlR, and expressed either WT *pqsE* or a *pqsE* mutant from pUCP18. Figure 3d shows that, in the presence of RhlR and C_4_HSL, WT PqsE and PqsE(D73A) enabled robust pyocyanin production, PqsE(E182W) and PqsE(E182W/S285W) promoted lower-level pyocyanin production, and PqsE(NI) did not drive production of pyocyanin. We note especially our findings with PqsE(E182W) and PqsE(E182W/S285W), shown in Fig. 3d; significantly higher levels of pyocyanin were produced in this context than from the strains shown in Fig. 3b. The key difference is that *rhlR* was expressed from its native promoter in Fig. 3b, while it was overexpressed from the pBAD promoter in Fig. 3d. The latter enabled high-level *rhlR* expression in the absence of the upstream LasR/LasI regulators (see Introduction and also Fig. S3b,c). We hypothesize that impairment in the PqsE-RhlR:C_4_HSL interaction can be overridden in the case of PqsE(E182W) and PqsE(E182W/S285W) by increasing the concentration of RhlR:C_4_HSL. However, proper interaction with PqsE α-helix 5 is required. Thus, overexpression of RhlR:C_4_HSL does not restore pyocyanin production when the PqsE(NI) variant is present (Fig. 3d). To confirm this supposition, we performed the RhlR-PqsE affinity purification assessment under varying concentrations of RhlR:mBTL. Indeed, increasing the concentration of RhlR:mBTL promoted increased complex formation with WT PqsE, PqsE(E182W) and PqsE(E182W/S285W), but not with PqsE(NI) (Fig. S4b). Collectively, these results indicate that the inhibitor mimetic mutations weaken the PqsE-RhlR interaction, while the NI alteration entirely blocks PqsE interaction with RhlR. Furthermore, the PqsE-RhlR interaction, relying on the α-helix 5 of PqsE, drives pyocyanin production *in vivo*.

### PqsE enhances the affinity of RhlR for promoter DNA.

**FIG 4:**
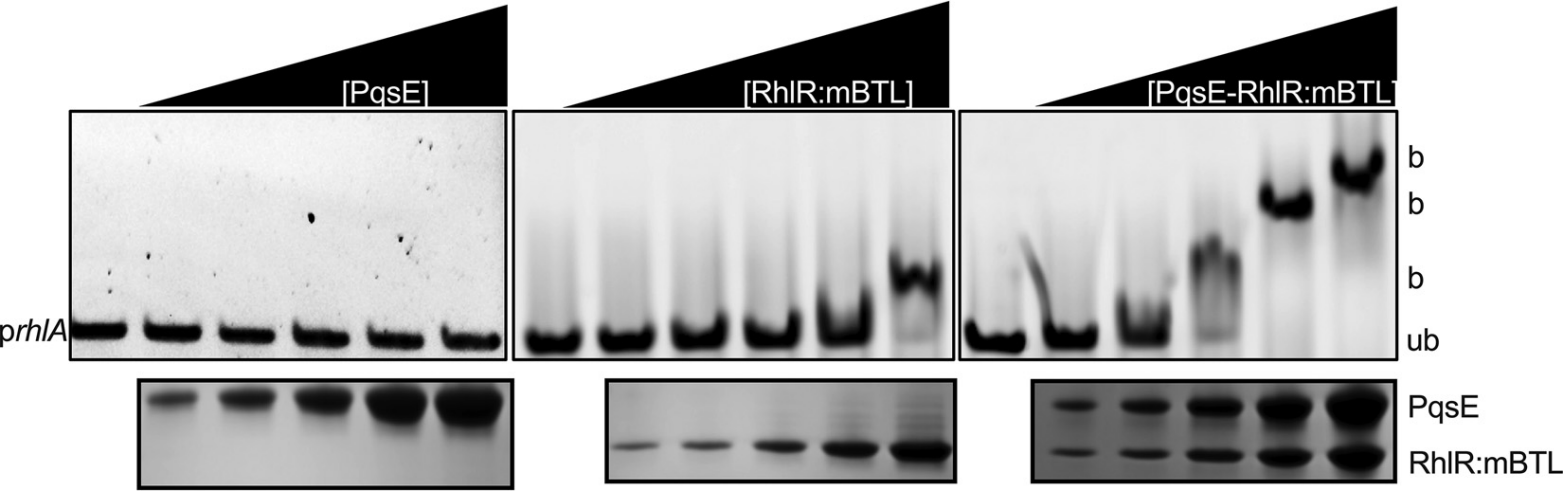
PqsE enhances RhlR:mBTL affinity for promoter DNA. EMSA of the *rhlA* promoter with purified PqsE (left), RhlR:mBTL (middle), and PqsE-RhlR:mBTL (right). “Ub” signifies DNA probe unbound by protein and “b” signifies DNA probe bound by protein. SDS-PAGE showing protein levels in the samples is provided below the corresponding EMSA results.

Given that PqsE can increase RhlR:C_4_HSL-dependent transcription in the E. coli reporter system and that PqsE-RhlR:C_4_HSL complex formation correlates with pyocyanin production levels *in vivo*, as mentioned, we hypothesized that the mechanism underlying PqsE-dependent regulation of RhlR:C_4_HSL is through the ability of PqsE to alter the affinity of RhlR:C_4_HSL for promoter DNA. To test this supposition, we used electrophoretic mobility shift assays (EMSA) to assess the DNA-binding affinity of RhlR:mBTL compared to that of RhlR:mBTL in complex with PqsE. In the EMSAs, we used a *rhlA* promoter fragment identical to the one we employed above in the E. coli p*rhlA-luxCDABE* reporter assays. PqsE does not possess a DNA-binding motif and we can find no evidence for PqsE binding to DNA in the absence of RhlR:mBTL (Fig. 4). When RhlR:mBTL was bound to PqsE, its affinity for *rhlA* promoter DNA increased ∼5-fold compared to that of RhlR:mBTL alone (Fig. 4). These results support our hypothesis that PqsE enhances holo-RhlR binding to promoter DNA.

### RNA-seq analyses of strains harboring variant PqsE proteins reveal distinct regulons responsive to the PqsE enzymatic and RhlR-interaction functions.

**FIG 5:**
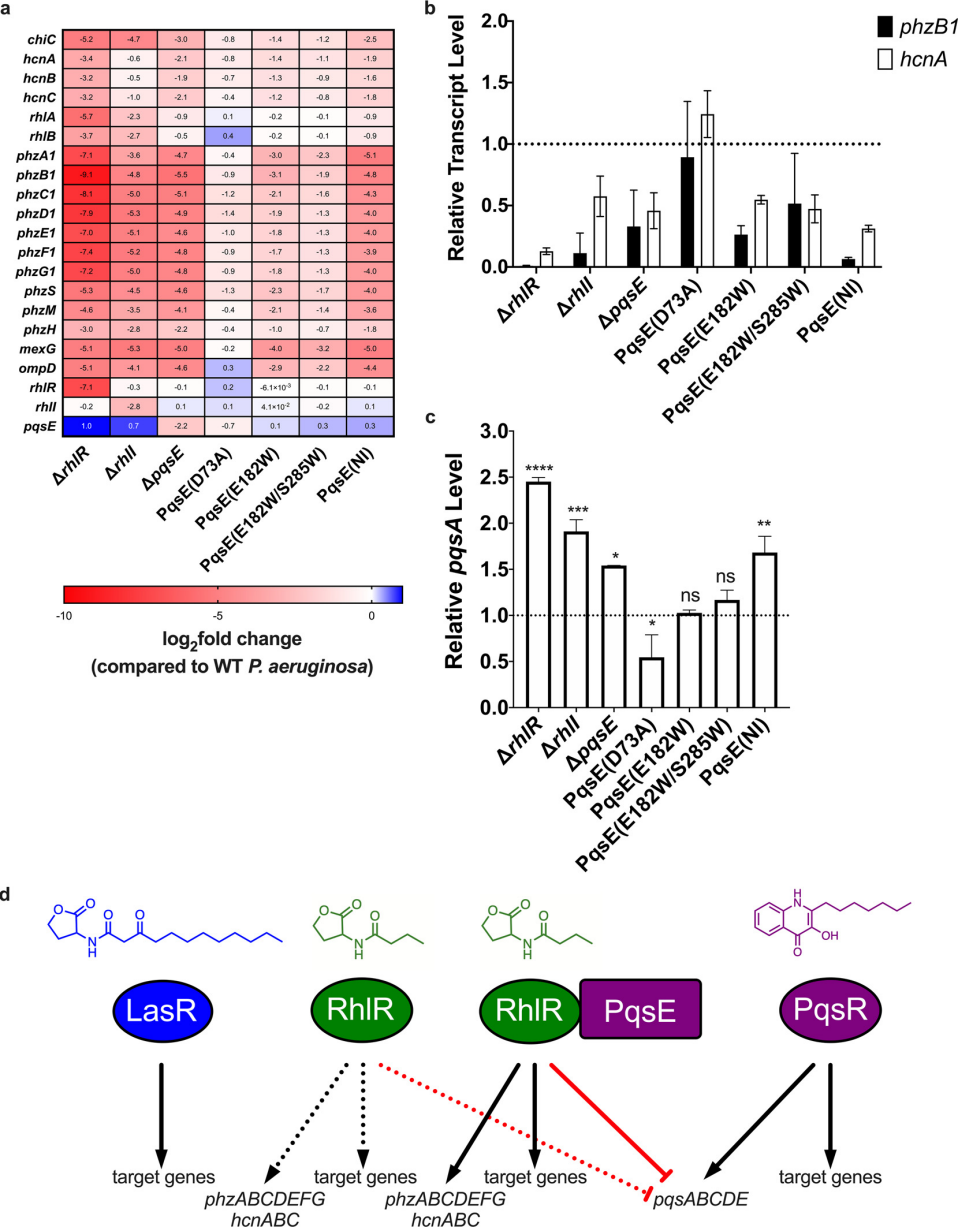
Genes regulated by the PqsE-RhlR:C_4_HSL interaction and PqsE catalytic function reveal dual roles for PqsE in virulence factor production. (a) RNA-seq heat map of virulence factor gene expression in strains lacking *rhlR*, *rhlI*, or *pqsE*, or expressing *pqsE* mutants from the chromosome, compared to expression levels from WT P. aeruginosa, shown as log_2_ fold-change; 0 = no change, <0 = downregulated genes (colored red), >0 = upregulated genes (colored blue). Data are representative of 2 independent RNA-seq experiments. For the full data set, see Table S1 in the supplemental material. (b) Confirmation of the regulation of the target genes *phzB1* (black) and *hcnA* (white) from strains in panel a using qRT-PCR. The dotted line represents the output of the strain carrying WT PqsE. Bars represent 3 biological replicates. Two technical replicates were performed and averaged for each gene in each biological replicate. Error bars represent standard deviations of the means of biological replicates. (c) Relative *pqsA* transcript levels in the designated P. aeruginosa strains. The dotted line represents the output of the strain carrying WT PqsE. Data are representative of 2 independent RNA-seq experiments. Unpaired *t* tests compared expression levels from each strain to that from the strain carrying WT PqsE. *P* values: ns, ≥ 0.05; *, <0.05; **, <0.01; ***, <0.001; ****, <0.0001. (d) A model showing the putative role of the PqsE-RhlR complex in P. aeruginosa along with major QS regulators and key target genes. Positive and negative regulation are shown with black arrows and red bars, respectively. As shown here, PqsE binding to RhlR enhances RhlR binding to DNA and increases positive regulation of target genes. RhlR repressor function is also enhanced by the interaction with PqsE. PqsE-dependent enhancement is depicted by the solid lines extending from the PqsE-RhlR complex to the target genes. The dotted lines extending from RhlR show basal regulatory function that does not require interaction with PqsE.

To determine the consequences of PqsE binding to RhlR:C_4_HSL on the regulation of gene expression, we performed RNA-seq analyses using P. aeruginosa strains harboring WT *pqsE*, *pqsE(D73A)*, *pqsE(E182W)*, *pqsE(E182W/S285W)*, and *pqsE(NI)* inserted at the native *pqsE* locus. As controls, we performed the same analyses on Δ*rhlR*, Δ*rhlI*, and Δ*pqsE* P. aeruginosa strains. This set of control strains allowed us to verify the entire RhlR regulon, and moreover, assess the reliance of RhlR-activated target genes on RhlI and on PqsE. Comparison of the output from WT P. aeruginosa to that of strains harboring PqsE variants defective in interaction with RhlR revealed that this interaction is crucial for proper control of gene expression *in vivo*. Indeed, we expected a largely shared regulon among the PqsE variants because PqsE-RhlR complex formation is disrupted to different extents by the different variants. Furthermore, comparing the output from the strain carrying the catalytically defective PqsE(D73A) variant with that from the strain carrying the noninteracting PqsE(NI) variant revealed the subset of RhlR-controlled genes which depend on PqsE catalysis. The strain harboring PqsE(D73A) served as an important control because this PqsE variant interacts with RhlR similarly to WT PqsE. Thus, PqsE (D73A) functions similarly to WT PqsE in its regulation of RhlR. We lay out the findings supporting these assertions here:

**(i) The PqsE-RhlR:C_4_HSL interaction regulon.** Overall, the RNA-seq revealed that a largely shared regulon is controlled by RhlR, RhlI, PqsE, PqsE(E182W), PqsE(E182W/S285W), and PqsE(NI). Figure 5a shows select data, the full data set is in Table S1. There are some key differences: P. aeruginosa harboring PqsE variants that impair complex formation with RhlR:C_4_HSL displayed altered transcriptional regulation of RhlR:C_4_HSL-dependent genes, the magnitudes of which correlated with the severity of their defects in PqsE-RhlR:C_4_HSL complex formation. To represent the differences, we describe the results for the RhlR:C_4_HSL-regulated *phzB1* gene which encodes a protein involved in phenazine biosynthesis. Figure 5a shows that deletion of *rhlR*, *rhlI*, and *pqsE* resulted in 597-, 29-, and 47-fold decreases in *phzB1* expression, respectively. In contrast, the strain harboring PqsE(D73A) showed almost no change in *phzB1* expression compared to the WT. PqsE(D73A) interacts like WT PqsE with RhlR, indicating that PqsE catalytic activity is dispensable for proper control of *phzB1*. P. aeruginosa strains carrying PqsE(E182W) and PqsE(E182W/S285W) exhibited decreased levels of *phzB1* expression compared to the strain with WT PqsE (8- and 4-fold reductions, respectively), tracking with their impaired but not abolished interactions with RhlR:mBTL *in vitro*. Finally, P. aeruginosa carrying PqsE(NI) displayed a 32-fold decrease in *phzB1* expression compared to the WT; indeed, an expression level similar to the strain lacking *pqsE*. These trends were consistent across all RhlR-dependent targets (Fig. 5a) and were confirmed by reverse transcription-quantitative PCR (qRT-PCR) for *phzB1* and *hcnA*, the latter of which encodes hydrogen cyanide synthase, another virulence factor (Fig. 5b). We highlight the results for other well-studied virulence genes in Fig. 5a and provide the complete RNA-seq data set in Table S1. Collectively, the results indicate that the PqsE effect on RhlR/RhlI-dependent transcriptional regulation is related to the ability of PqsE to bind to RhlR:C_4_HSL and enhance its affinity for promoter DNA.

**(ii) The PqsE catalytic activity regulon.** P. aeruginosa strains carrying PqsE(D73A) showed no or only modest changes in RhlR:C_4_HSL-regulated target genes compared to P. aeruginosa carrying WT PqsE (Fig. 5a and b). Thus, PqsE catalytic activity is not a major requirement for proper control of genes by RhlR:C_4_HSL. However, several transcripts were notably dysregulated in the strain with PqsE(D73A) compared to the strains with our other PqsE variants. We highlight *pqsA* as our representative in Fig. 5c and include the complete data set in Table S1. These analyses allow us to distinguish the role of PqsE catalysis from that of PqsE interaction with RhlR:C_4_HSL in controlling virulence genes and possibly other processes. Regarding *pqsA*, as anticipated, compared to WT P. aeruginosa, increased expression occurred in the Δ*rhlR* and Δ*rhlI* strains, consistent with RhlR:C_4_HSL repression of *pqsA* transcription (Fig. 5c, 30). In contrast, the presence of the PqsE(D73A) variant caused a reduction in *pqsA* expression, indicating that PqsE-directed catalysis is required for activation of *pqsA* expression (Fig. 5c). The strains with the PqsE(E182W) and PqsE(E182W/S285W) variants showed no differences in *pqsA* expression compared to the strain with WT PqsE, while the Δ*pqsE* strain and the strain with PqsE(NI) showed modest increases in expression (Fig. 5c). The Δ*pqsE* strain is fully defective in both catalysis and interaction, the strains with PqsE(E182W) and PqsE(E182W/S285W) are partially defective in both functions, and the strain with PqsE(NI) is partially defective in catalysis and fully defective in interaction. We interpret the respective PqsE effects on *pqsA* expression to be products of differences in the balance between the loss of PqsE-RhlR:C_4_HSL-mediated repression and the loss of PqsE-catalysis-mediated activation. For example, in the strain containing PqsE(D73A), *pqsA* expression is skewed toward repression due to interaction of PqsE(D73A) with RhlR:C_4_HSL. In contrast, in the strain containing PqsE(NI), *pqsA* expression is skewed toward activation due to the partial catalytic activity displayed by the PqsE(NI) variant. We expect that if we had a PqsE(NI) variant that possessed WT catalytic activity, it would exhibit a phenotype identical to that of the Δ*rhlR* mutant; i.e., maximal *pqsA* expression because expression would be subject to full PqsE-catalysis-mediated activation, but there would be no repression from PqsE interaction with RhlR:C_4_HSL. Finally, Fig. 5c shows that PqsE-RhlR:C_4_HSL-dependent repression of *pqsA* largely masks *pqsA* activation due to PqsE-mediated catalysis. We therefore infer that the dominant role of PqsE in regulation of *pqsA* expression stems from its interaction with RhlR:C_4_HSL (Fig. 5d).

## DISCUSSION

PqsE and RhlR, core components of the P. aeruginosa QS system, coregulate many genes, including those involved in the production of the virulence factor pyocyanin. It was previously shown that PqsE, an enzyme, binds to RhlR to regulate RhlR function as a transcription factor (37). Here, using a set of P. aeruginosa strains containing catalytic, inhibitor mimetic, and non-RhlR-interacting PqsE variants, we characterized the distinct roles PqsE plays in regulating RhlR DNA binding, RhlR-driven pyocyanin production, and RhlR-controlled gene expression. We find that PqsE control of RhlR function is independent of its enzymatic activity, since the PqsE(D73A) variant, which is catalytically inactive, interacted like WT PqsE with RhlR *in vitro* (Fig. 1b). Likewise, P. aeruginosa strains harboring the PqsE(D73A) variant produced pyocyanin at levels similar to WT P. aeruginosa (Fig. 3a to d). However, P. aeruginosa strains harboring PqsE variants which mimic the inhibitor-bound state of PqsE, PqsE(E182W) and PqsE(E182W/S285W), do not produce pyocyanin (Fig. 3a to d); moreover, these PqsE variants exhibit impaired interaction with RhlR *in vitro* (Fig. 1b). These results indicate that interaction between PqsE and RhlR *in vivo* is required to activate the expression of genes involved in pyocyanin production (Fig. 5d). Interestingly, PqsE residues E182W and S285W are buried in the catalytic pocket of PqsE and therefore cannot be part of the interaction interface. In contrast, the PqsE variant, PqsE(NI), which has three surface arginine residues altered to alanines, possesses catalytic activity, does not enable production of pyocyanin, and does not interact with RhlR *in vitro*. Thus, we suspect that PqsE residues R243, R246, and R247 are involved in forming the surface by which PqsE interacts with RhlR.

RhlR is a ligand-dependent, LuxR-type receptor, and the mechanism of PqsE-dependent regulation of RhlR is apparently unique among this family of receptors. To the best of our knowledge, PqsE is the first protein identified as a binding partner which enhances the affinity of a LuxR-type receptor for DNA (Fig. 4). Existing structures of PqsE guided the mutagenesis approach outlined in the present work. However, no structure of RhlR exists and we have not yet identified RhlR variants which disrupt the interaction with PqsE. Furthermore, the conformational changes RhlR undergoes upon binding to PqsE have not been defined and will be key to understanding the molecular basis for PqsE-enhancement of RhlR recognition of promoter DNA. Structural studies of catalytically inactive PqsE that can fully or partially interact with RhlR, alone and in complex with RhlR, would be informative for discovering how changes in the catalytic pocket can affect the PqsE-RhlR interface. We are attempting to obtain such structures now. Comparisons of structures of other LuxR-type receptors, without DNA and bound to DNA, have revealed that the C-terminal DNA-binding domains (DBD) can adopt multiple conformations relative to the N-terminal ligand-binding domains (LBD) via a flexible linker region, and such rearrangement is key to DNA binding (40–43). The inhibitor-bound state of one particular LuxR-type receptor, CviR from Chromobacterium violaceum, showed that the DBD adopted a “closed” conformation, such that the helices responsible for making contact with DNA were situated in a configuration that made DNA binding impossible (44). This structure also pointed to flexibility in the linker region connecting the LBD to the DBD as driving receptor affinity for DNA. We hypothesize that the DBD of RhlR:C_4_HSL, when not bound to PqsE, adopts different conformations in solution, but is biased toward a “closed” non-DNA-binding confirmation. Such a scenario would track with the known low affinity RhlR:mBTL exhibits for DNA *in vitro*. For example, RhlR:mBTL has a *K*_d_ (dissociation constant) = 30 nM for the so-called *rhl* box sequence, whereas LasR:3OC_12_HSL binds the analogous *las* box with a *K*_d_ of 11 pM (16, 34). Perhaps, upon binding to PqsE *in vivo*, RhlR:C_4_HSL adopts the “open” conformation and becomes capable of higher-affinity DNA binding. It is interesting that RhlR possesses a mechanism to increase its intrinsic affinity for promoter DNA. By interacting with PqsE at some promoters but not others, RhlR could expand the range of levels with which it activates target gene expression. We anticipate that additional regulatory mechanisms could control PqsE-RhlR:C_4_HSL complex formation to further modulate RhlR-dependent gene expression.

In addition to establishing the role of PqsE in regulating RhlR-dependent transcription, our RNA-seq analyses provided initial insights into the role PqsE-driven catalysis plays in gene regulation, presumably through its proposed function of converting 2-ABA-CoA to 2-ABA. Indeed, our data (Fig. 5c) show that we can uncouple the enzymatic and nonenzymatic functions of PqsE. As shown in the results, *pqsA* expression is activated by PqsE catalytic activity and repressed by PqsE interaction with RhlR:C_4_HSL (Fig. 5d). To reconcile these findings, we hypothesize that PQS biosynthesis is enhanced as a consequence of PqsE enzyme function, despite PqsE not being absolutely required for PQS synthesis. Once PQS is made, it binds to PqsR, which launches the autoinduction feedback loop that boosts *pqsABCDE* expression. Thus, autoinduction increases PqsE production and drives increased PqsE-RhlR:C_4_HSL complex formation, assuming that RhlR:C_4_HSL is not limiting. PqsE-RhlR:C_4_HSL represses *pqsABCDE* expression, an activity that relies on the nonenzymatic function of PqsE (Fig. 5d). It is possible that PqsR:PQS and PqsE-RhlR:C_4_HSL compete for binding to the *pqsABCDE* promoter. If so, the outcome of this competition would be dictated by the amount and affinity of substrate available to PqsE for catalysis to funnel precursors into the PQS biosynthetic pathway versus the amount and affinity of RhlR:C_4_HSL for PqsE to bind. Dual and opposing regulation of *pqsABCDE* by the two distinct PqsE functions could control the timing and the strength of expression of the Rhl- and PqsR-controlled regulons in the P. aeruginosa QS hierarchy.

Our characterization of the PqsE-RhlR interface provides the molecular basis for regulation of RhlR by PqsE; moreover, it demonstrates that PqsE-RhlR:C_4_HSL complex formation, not PqsE-directed catalysis, is primarily responsible for the transcriptional activation of genes involved in pyocyanin production and other traits important for pathogenesis. Thus, the PqsE-RhlR interface discovered here represents a new candidate for targeting with small molecule modulation. Compounds that disrupt the PqsE-RhlR interaction should suppress virulence. Additionally, the PqsE-RhlR interaction is not required for growth, as strains harboring PqsE(NI) did not exhibit growth defects. Thus, potential inhibitors of the PqsE-RhlR interaction should not be as vulnerable to resistance-promoting mutations as targets of traditional antibiotics.

## MATERIALS AND METHODS

### Strain and plasmid construction.

Standard cloning and molecular biology techniques were used to generate E. coli and P. aeruginosa overexpression plasmids. Introduction of genes encoding PqsE variants onto the P. aeruginosa chromosome was achieved using previously published protocols (34, 45). In brief, the entire *pqsE* coding sequence, in addition to 500 bp of upstream and downstream DNA, were amplified, digested, ligated into the pEXG2 suicide vector, transformed into E. coli SM10 λ*pir*, and conjugated into the appropriate P. aeruginosa strain. All strains and plasmids used in this study are shown in Table S2. Primers are shown in Table S3 in the supplemental material.

### Affinity purification pulldown.

Overnight cultures of E. coli strains carrying overexpression vectors for producing variant 6x-His-PqsE proteins or RhlR were diluted 1:100 and grown at 37°C with shaking to an OD_600_ = 1.0. Protein production was induced by the addition of 1 mM isopropyl-β-d-thiogalactopyranoside IPTG. Ten μM mBTL was added to the strain producing RhlR. Identical conditions were used for production of RhlR* in E. coli except that mBTL was omitted. After 4 h, cells were pelleted by centrifugation and the pellets frozen until lysis. Lysis buffer (20 mM Tris-HCl, 150 mM NaCl [pH 8.0]) was added in proportion to the pellet size (100 μL/5 mL culture). Resuspended pellets were transferred to microcentrifuge tubes and lysed via sonication, followed by centrifugation at 15,000 × *g* at 4°C for 20 min. Equal amounts of supernatant fractions from PqsE- and RhlR-containing cells were combined, and 10 μL was saved for input assessment. Invitrogen MagneHis Ni-Particle beads (20 μL per sample) were washed with lysis buffer and resuspended in lysis buffer at 100 μL/sample, followed by mixing with the above protein samples for 1 h at 4°C with inversion. Samples were subjected to brief centrifugation at 250 × *g*, placed on a magnetic rack, and the clarified supernatants aspirated. Samples were washed three times with lysis buffer and 6x-His-protein was eluted with two washes of 20 μL of 1 M imidazole. Eluted protein was mixed with 2× sample buffer and loaded onto SDS-PAGE gels. Gels were stained with Coomassie brilliant blue and imaged on a Bio-Rad EZ-Doc gel imager.

### PqsE-RhlR coupled p*rhlA-luxCDABE* assay.

*pqsE* and *rhlR* were expressed from the *lac* promoter and the pBAD promoter, respectively, in an E. coli strain containing the p*rhlA*-*luxCDADE* fusion. C_4_HSL (Cayman Chemical) was supplied at 500 nM. The protocol has been described previously (37).

### Enzyme assay measuring MU-butyrate hydrolysis.

PqsE enzymatic activity was measured as described previously, with modifications (37). Briefly, PqsE proteins were incubated at 125 nM with 2 μM 4-methylumbelliferyl butyrate (MU-butyrate) in assay buffer (50 mM Tricine, 0.01% Triton X-100 [pH 8.5]). Fluorescence (ex: 360 nm, em: 450 nm) was immediately measured in a plate reader (BioTek) at 30 s intervals for 30 min. The hydrolysis rate was calculated over the initial 3 min of the reaction.

### PqsE and PqsE-RhlR enzyme kinetics.

Samples of PqsE and PqsE in complex with RhlR:mBTL were prepared via pulldown on Ni-NTA resin as described previously (37). The eluted samples were analyzed by SDS-PAGE along with a dilution series of purified PqsE protein to determine the concentration of PqsE in each sample (Fig. S2b). The PqsE and PqsE-RhlR:mBTL samples were diluted to a final assay concentration of 125 nM PqsE and placed in wells of an opaque 384-well plate (Corning); subsequently, MU-butyrate (Sigma) was added at 10 μM and a series of 2-fold dilutions was made. Fluorescence was immediately measured at 30 s intervals for 20 min, and initial hydrolysis rates were determined over the first 3 min of the assay. Results are reported as RFU/min (RFU, Relative Fluorescence Units). The *K*_m_ was determined for PqsE and for the PqsE-RhlR:mBTL complex using Prism 9.0 software. The melting temperature (*T_m_*) of each purified PqsE protein was determined as described previously (37).

### Pyocyanin assay.

Overnight cultures were diluted 1:100 in 3 mL LB containing 400 μg/mL carbenicillin in the cases of strains harboring plasmid-borne *pqsE* genes on pUCP18. Dilutions were into 3 mL LB in cases of strains harboring *pqsE* alleles carried on the chromosome. All strains were grown and pyocyanin was measured as previously described (34).

### *P. aeruginosa rhlR* overexpression.

Overnight cultures of P. aeruginosa carrying pBAD-*rhlR* were diluted 1:100 in LB with carbenicillin (400 μg/mL) and grown at 37°C with shaking for 2 h, until OD_600_ = 1.0. *rhlR* expression was induced by the addition of 0.1% arabinose and 10 μM C_4_HSL, followed by 4 h growth. The cells were pelleted at 8,600 × *g* for 5 min, and the pellets frozen at −80°C until lysis. Cells were resuspended in lysis buffer (20 mM Tris-HCl, 150 mM NaCl, 20 mM imidazole [pH 8.0]) and lysed via sonication. Suspensions were subjected to centrifugation at 18,000 × *g* at 4°C for 30 min. Supernatants were collected, mixed 1:1 with 2× sample buffer, and loaded onto SDS-PAGE (Bio-Rad). Protein was transferred to polyvinylidene difluoride (Bio-Rad) membranes at 100 V for 50 min. Blocking was performed with 1× phosphate-buffered saline with Tween 20 (PBST) and 5% milk. Primary α-RhlR and α-PqsE polyclonal antibodies from rabbit (Cambridge Antibodies) were incubated with membrane overnight using 1:1,000 dilutions; this was followed by incubation with goat α-rabbit IgG2b antibody which was cross adsorbed with secondary antibody conjugated with horseradish peroxidase (ThermoFisher) for 1 h at a 1:10,000 dilution. All antibody solutions were made in PBST with 5% milk.

### Protein production and purification.

Two separate E. coli cultures, one carrying the plasmid containing the gene encoding WT *6x-His-pqsE* or the gene specifying a *6x-His-pqsE* mutant and the other carrying *rhlR* under the control of the T7 promoter, were grown overnight in LB containing kanamycin (50 μg/mL for *pqsE* expression) or ampicillin (100 μg/mL for *rhlR* expression). Cultures were diluted 1:100 in fresh medium and grown at 37°C for 4 h. At OD_600_ = 1.0, protein production was induced by the addition of 1 mM IPTG and the cultures were incubated at room temperature (RT) for another 4 h. 100 μM mBTL was added to the cultures of cells producing RhlR. Cells were harvested at 8,600 × *g* and the pellets were frozen until protein purification. Due to differences in protein production levels between PqsE and RhlR, 4 L of cultures producing untagged RhlR were grown for every 1 L of culture producing 6x-His-PqsE. Pellets were resuspended and lysed as described for the affinity purification pulldown. Supernatants were mixed and incubated with Ni-NTA resin (New England Biolabs) for 2 h. Complexes were eluted with lysis buffer containing 250 mM imidazole and subjected to separation on a Superdex-200 column equilibrated with 20 mM Tris-HCl and 150 mM NaCl (pH 8.0). PqsE-RhlR:mBTL complex purity was assessed by SDS-PAGE.

### Electrophoretic mobility shift assay.

PqsE and RhlR:mBTL concentrations were standardized to their relative concentrations in the PqsE-RhlR:mBTL complex. EMSA reactions were comprised of 17 μL EMSA buffer (200 mM KCl, 50 mM Tris-HCl, 250 μg/mL bovine serum albumin, 50 mM NaCl, 5 nM EDTA, 5 μM MgCl_2_, 5 μM dithiothreitol [pH 8.0]), 2 μL of protein dilution, and 1 μL of 10 ng/μL *rhlA* promoter DNA. The reactions were initiated and incubated at 30°C for 15 min. One μL of Novex Hi-Density TBE 5× sample buffer (ThermoFisher) was mixed with 9 μL of the EMSA reaction and loaded on a 2.5% agarose gel made in 0.5× TB buffer. Electrophoresis was performed in 0.5× TB buffer at 150 V for 60 min followed by washing at RT with 50 mL of 0.5× TB for 15 min. Gels were stained with 50 mL 1× SYBR Gold in 0.5× TB buffer for 30 min at RT, washed with 50 mL of 0.5× TB buffer three times for 15 min, and visualized on a Bio-Rad EZ-Doc gel imager (ex: 495 nm, em: 537 nm). The increase in affinity was determined based on the amount of RhlR protein required to shift 100% of the *rhlA* promoter DNA in the EMSA.

### RNA extraction, sequencing, and data analysis.

Overnight cultures of P. aeruginosa strains were back diluted 1:100 in 25 mL and incubated for 5 h at 37°C with shaking. Cells were harvested by centrifugation and pellets were frozen until RNA extraction. Frozen pellets were resuspended in 800 μL TRIzol (ThermoFisher) and these preparations were added to ∼100 μL silica beads in screw-cap tubes. Samples were homogenized with a bead beater. A volume of 100 μL chloroform was added to each sample, the samples were shaken vigorously by hand for 15 s, and the preparations were subjected to centrifugation at 12,000 × *g* for 15 min at 4°C. Nucleic acid-containing fractions were transferred to a new microcentrifuge tube and 500 μL isopropanol was added. Samples were mixed briefly and subjected to centrifugation at 12,000 × *g* for 10 min at 4°C. The resulting supernatants were aspirated. Pellets were resuspended in 1 mL 70% ethanol and subjected to centrifugation at 10,000 × *g* for 5 min at 4°C, followed by aspiration of the ethanol. Pellets were air-dried until traces of ethanol had evaporated. Pellets were resuspended in 50 μL nuclease-free water and incubated briefly at 37°C for solubilization. DNA was depleted from 3 μg of each RNA sample using the TURBO DNase kit containing SUPERase-in RNase Inhibitor (Thermo Fisher) at 37°C for 30 min in 30 μl total reaction volumes. A volume of 3 μL DNase Inactivation slurry (Thermo Fisher) was added to each sample, followed by incubation at RT for 5 min with shaking. Samples were subjected to centrifugation at 10,000 × *g* for 2 min. A 25-μL volume of supernatant was transferred to a new microcentrifuge tube and these samples were frozen at −80°C until cDNA library preparation. cDNA libraries were made with the NEBNext Ultra II RNA Library Preparation Kit for Illumina according to the manufacturer’s protocol (New England Biolabs). AMPure XP purification beads (Beckman Coulter) were used at the ratios indicated by the manufacturer’s protocol, except for the final elution in which 0.8× of the manufacturer’s recommended elution volume was used. Paired-end libraries (50 bp × 30 bp) were sequenced on an Illumina NextSeq platform. Reads were quality-trimmed and any remaining adapters were removed by the bbduk function from BBtools v38.86 (https://sourceforge.net/projects/bbmap/), which required reads to be a minimum of 15 bp and the average read quality to be 20. Reads were mapped to the reference assembly CP034244.1 (P. aeruginosa UCBPP-PA14) by BWA v.0.7.17 (46) and the number of reads spanning coding sequences was extracted using the multiBamCov function from Bedtools v.2.29.2 (47). Differentially expressed genes were identified by DESeq2 (48) in R V.4.4.1 (http://www.R-project.org). Genes with an adjusted *P* value of ≤ 0.5 were considered to be differentially expressed.

### RT-PCR.

One μg of DNA-depleted samples of mRNA was incubated with random hexamers (Integrated DNA Technologies) at 65°C for 5 min and the mixtures transferred to ice. cDNA was prepared with a SuperScript III Reverse Transcriptase kit (Invitrogen) in total reaction volumes of 20 μL. SYBR Select Master Mix (Applied Biosystems) was used for RT-PCR. Briefly, 2× SYBR Select was mixed with primers (200 nM final concentration) (Table S3) and 18 μl were aliquoted per well. Finally, 20-μL cDNA reactions were diluted 1:5 and 2 μL added per well. A 7500 Fast real-time PCR system (Applied Biosystems) and software (v2.3) were used for cycle threshold quantification and relative gene expression analysis.

### Data availability.

Sequencing data have been deposited at the NCBI Sequence Read Archive under the submission number SUB10815364 and the NCBI BioProject number PRJNA789860.
