# Supplementary material for: The PqsE-RhlR Interaction Regulates RhlR DNA Binding to Control Virulence Factor Production in Pseudomonas aeruginosa
Source: Microbiol Spectr. 2022 Jan 12;10(1):e02108-21. doi: 10.1128/spectrum.02108-21 (PMC8754118; doi:10.1128/spectrum.02108-21)
Supplement: SUPPLEMENTAL FILE 4 — Supplemental material. Download SPECTRUM02108-21_Supp_4_seq12.pdf, PDF file, 1.3 MB [file spectrum02108-21_supp_4_seq12.pdf]

SUPPLEMENTAL FIGURE LEGENDS

Figure S1. *WT PqsE and PqsE variants are produced at similar levels in the* E. coli *reporter strain.* Western blot analysis of *E. coli* reporter strains producing RhlR:C_4HSL or RhlR:C_4HSL together with WT PqsE or PqsE variants. 10 μg of protein from whole cell lysates were loaded per sample. PqsE and RhlR:C_4HSL were detected by PqsE- and RhlR-specific antibodies. Load represents total protein loaded into each lane for the corresponding strains.

Figure S2. *Thermal stabilities of PqsE and PqsE variants.* (a) Thermal stability profiles for purified 6x-His-PqsE proteins. First derivative plots of the raw fluorescence curves are shown for WT PqsE (black), PqsE(D73A) (gray), PqsE(E182W) (blue), PqsE(E182W/S285W) (purple), and PqsE(NI) (red). The peak of each curve is interpreted as the T_m of that variant. (b) Purified WT PqsE was added to the first 8 lanes of the gel in 2-fold dilution series. PqsE alone and PqsE-RhlR:mBTL eluted from the Ni-NTA resin are in the final two lanes, respectively. A standard curve generated from the dilution series was used to determine the concentration of PqsE in the final two samples.

Figure S3. *WT PqsE and variant PqsE production levels following chromosome or plasmid expression and corresponding pyocyanin output*. (a) Western blot analysis of PqsE variant protein production levels when the genes producing the designated PqsE variants were introduced into *P. aeruginosa* at the native *pqsE* locus. 10 μg of protein from whole cell lysates were loaded per sample. (b) Percent pyocyanin production relative to WT *P. aeruginosa* from the same strains shown in panel a. Bars represent 3 biological

replicates. Two technical replicates were performed and averaged for each biological replicate. Error bars represent standard deviations of the means of biological replicates. Unpaired *t*-tests compared pyocyanin production from each strain to that produced by *P. aeruginosa* carrying WT PqsE. P-values: ns ≥ 0.05, *** < 0.001. (c) Western blot analysis of RhlR:C_4HSL following expression of *rhlR* from its native promoter and from the pBAD promoter with (+) or without (-) 0.1% arabinose. 10 μg of protein from whole cell lysates were loaded per sample. Load represents total protein loaded into each lane for the corresponding strains.

Figure S4. *PqsE(NI) does not interact with constitutively activated or overexpressed RhlR.* (a) SDS-PAGE showing cell lysates before (Input) and after (Elution) affinity purification on Ni-NTA resin. Shown are WT and variant 6x-His-PqsE-containing lysates that had been combined with lysate containing RhlR*. (b) RhlR:mBTL-containing lysate was serially diluted 1:5 in 200 μL of lysis buffer and 50 μL of each dilution was combined with lysates containing WT PqsE, PqsE(E182W), PqsE(E182W/S825W), and PqsE(NI).

SUPPLEMENTAL TABLES

Table S1. *RNA-seq data set.* This table shows the $\log_2$fold change of all transcripts for all strains used in this study compared to WT *P. aeruginosa*.

Table S2. *Strains and plasmids used in this study*. This table lists the names, sources, and genotypes of all strains used in this study.

Table S3. *Primers used in this study*. This table lists the names, sequences (5' to 3'), and uses of all primers employed in this study.

REFERENCES

1. Mukherjee S, Moustafa DA, Stergioula V, Smith CD, Goldberg JB, Bassler BL. The PqsE and RhlR proteins are an autoinducer synthase-receptor pair that control virulence and biofilm development in Pseudomonas aeruginosa. Proc Natl Acad Sci U S A. 2018/09/19. 2018;115: E9411–E9418. doi:10.1073/pnas.1814023115.

2. McCready AR, Paczkowski JE, Cong J-P, Bassler BL. An autoinducer-independent rhlr quorum-sensing receptor enables analysis of rhlr regulation. PLoS Pathog. 2019;15. doi:10.1371/journal.ppat.1007820

3. Mukherjee S, Moustafa DA, Stergioula V, Smith CD, Goldberg JB, Bassler BL. The RhlR quorum-sensing receptor controls Pseudomonas aeruginosa pathogenesis and biofilm development independently of its canonical homoserine lactone autoinducer. PLoS Pathog. 2017;13 doi: https://doi.org/10.1371/journal.ppat.1006504

4. Taylor IR, Paczkowski JE, Jeffrey PD, Henke BR, Smith CD, Bassler BL. Inhibitor Mimetic Mutations in the Pseudomonas aeruginosa PqsE Enzyme Reveal a Protein–Protein Interaction with the Quorum-Sensing Receptor RhlR That Is Vital for Virulence Factor Production. ACS Chem Biol. 2021;16: 740–752. doi:10.1021/acschembio.1c00049

Figure S1

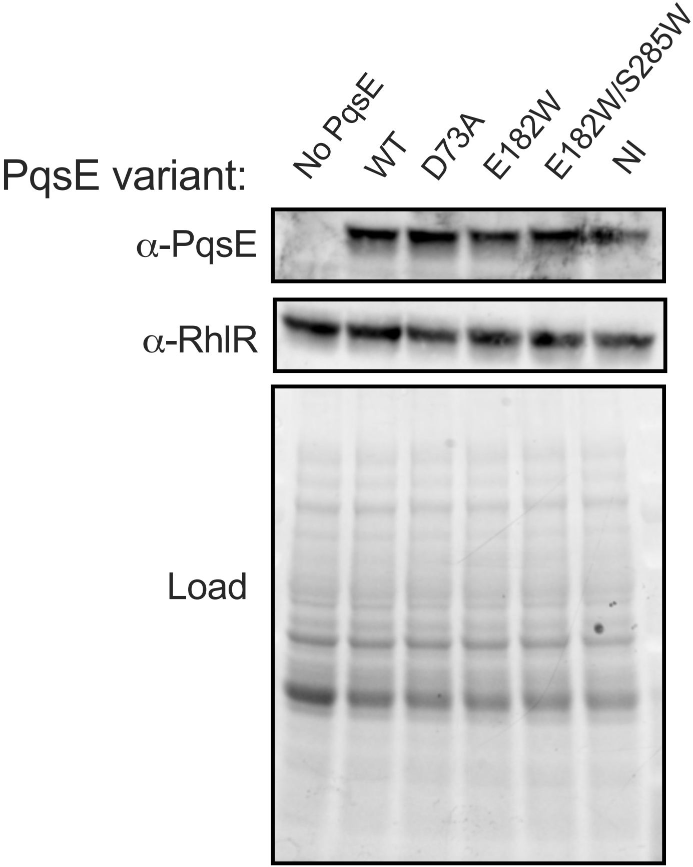
PqsE variant:
No PqsE
WT
D73A
E182W
E182W/S285W
NI
α-PqsE
α-RhlR
Load

Figure S2

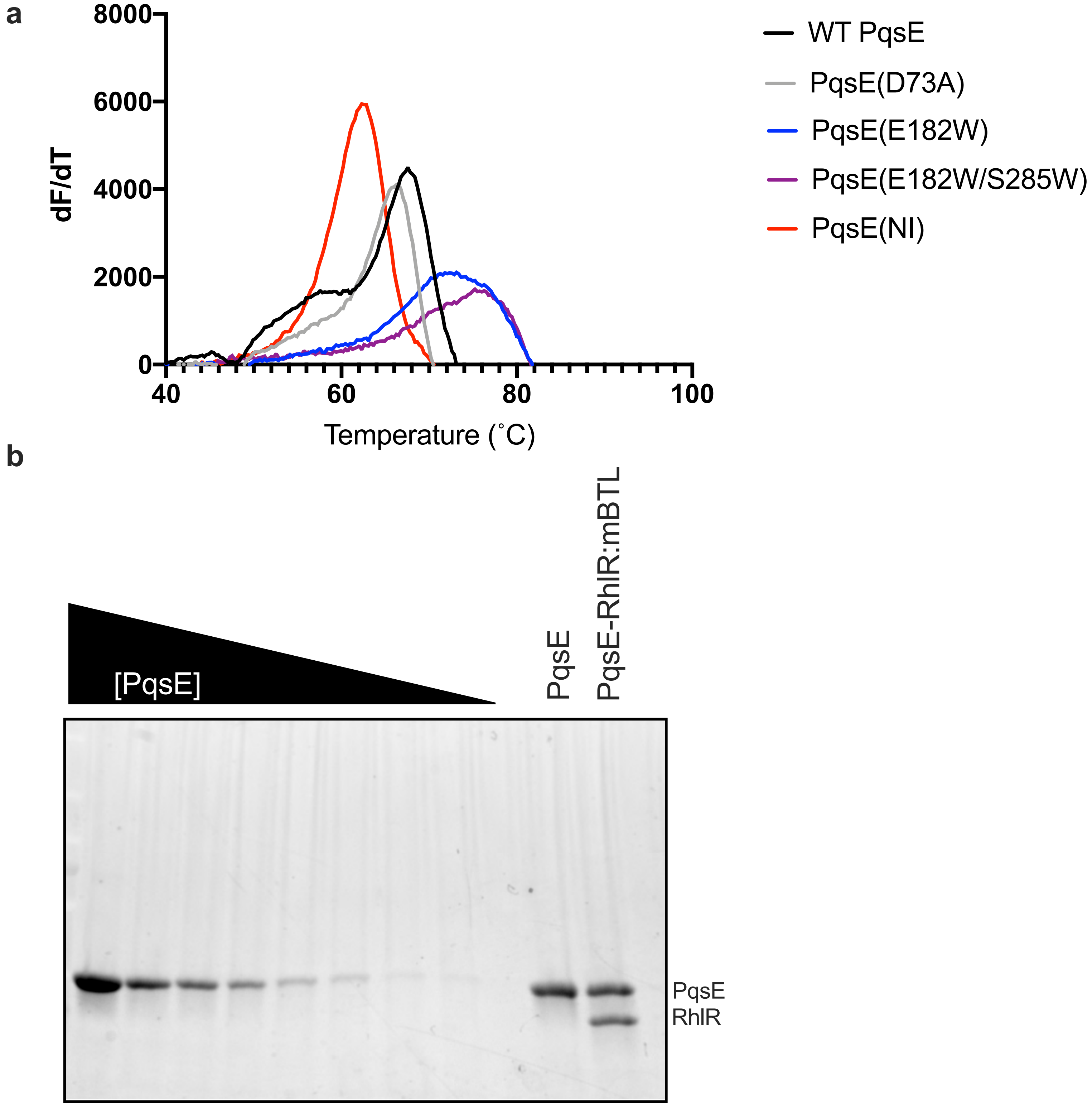
a
dF/dT
8000
6000
4000
2000
0
40
60
80
100
Temperature (˚C)
WT PqsE
PqsE(D73A)
PqsE(E182W)
PqsE(E182W/S285W)
PqsE(NI)
b
[PqsE]
PqsE
PqsE-RhlR:mBTL
PqsE
RhlR

Figure S3

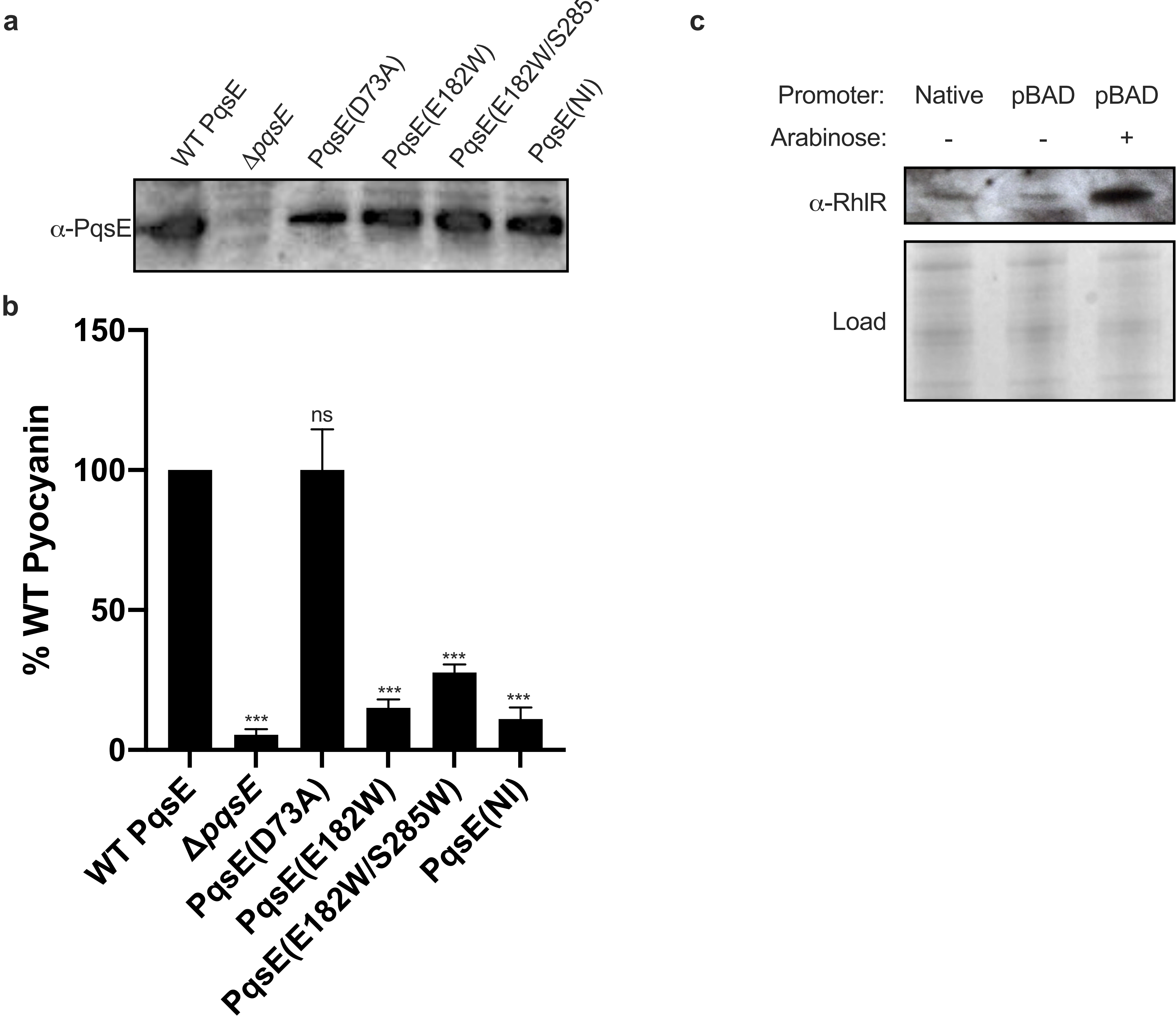
a
WT PqsE
ΔpqsE
PqsE(D73A)
PqsE(E182W)
PqsE(E182W/S285W)
PqsE(NI)
α-PqsE
b
% WT Pyocyanin
150
100
50
0
ns
***
***
***
***
WT PqsE
ΔpqsE
PqsE(D73A)
PqsE(E182W)
PqsE(E182W/S285W)
PqsE(NI)
c
Promoter: Native pBAD pBAD
Arabinose: - - +
α-RhlR
Load

Figure S4

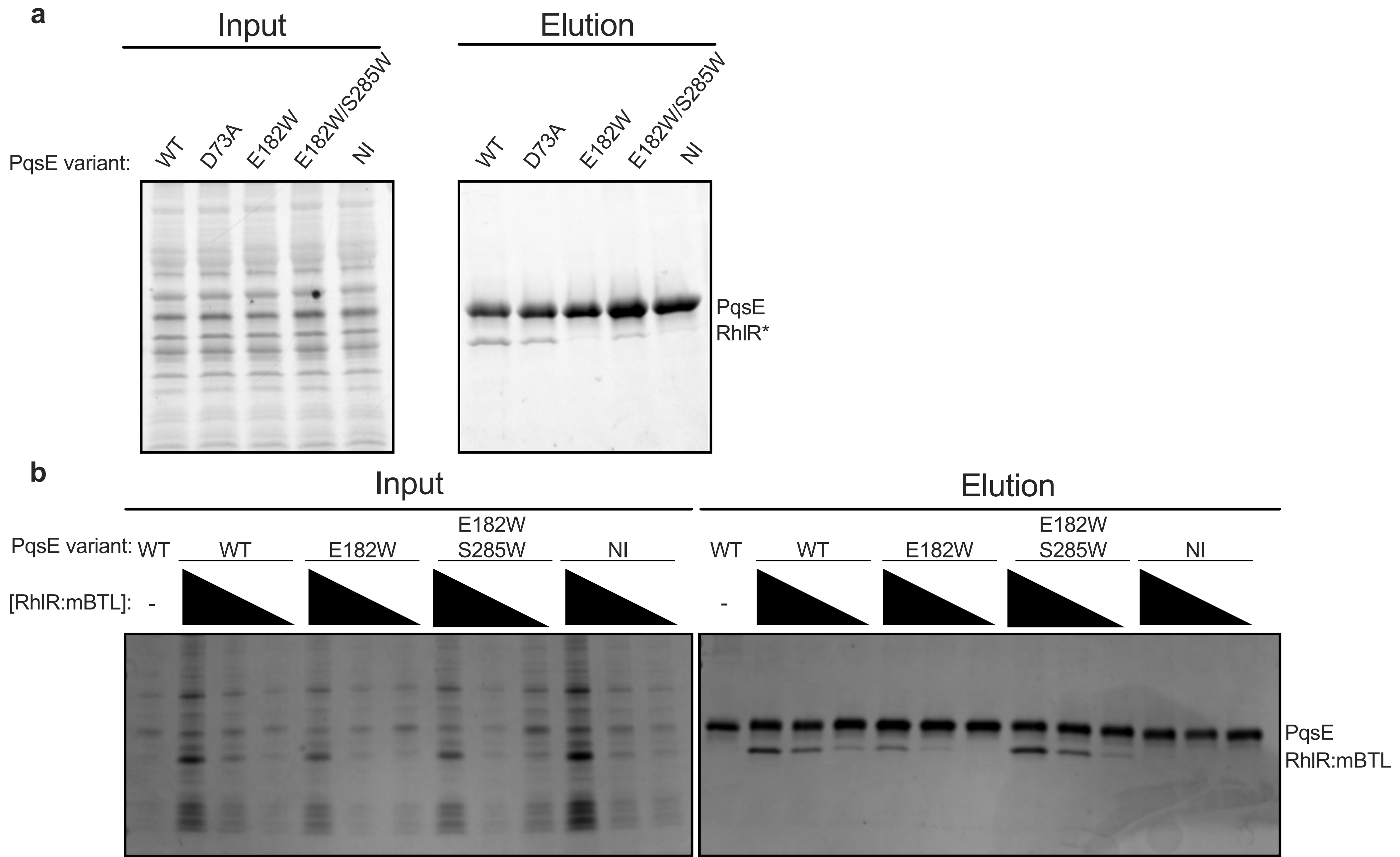
a
Input
Elution
PqsE variant:
WT
D73A
E182W
E182W/S285W
NI
PqsE
RhlR*
b
Input
Elution
PqsE variant:
WT
E182W
E182W
S285W
NI
[RhlR:mBTL]:
-
PqsE
RhlR:mBTL
